# HPMA Copolymer Mebendazole Conjugate Allows Systemic Administration and Possesses Antitumour Activity In Vivo

**DOI:** 10.3390/pharmaceutics14061201

**Published:** 2022-06-04

**Authors:** Martin Studenovský, Anna Rumlerová, Jiřina Kovářová, Barbora Dvořáková, Ladislav Sivák, Libor Kostka, Daniel Berdár, Tomáš Etrych, Marek Kovář

**Affiliations:** 1 Institute of Macromolecular Chemistry, Czech Academy of Sciences, Heyrovského nám. 2, 16206 Prague, Czech Republic; annarumlerova@centrum.cz (A.R.); kostka@imc.cas.cz (L.K.); etrych@imc.cas.cz (T.E.); 2 Institute of Microbiology, Czech Academy of Sciences, Vídeňská 1083, 14220 Prague, Czech Republic; jirina.kovarova@biomed.cas.cz (J.K.); dvorakova@bio-port.cz (B.D.); l.sivak@yahoo.com (L.S.); daniel.berdar@ibt.cas.cz (D.B.)

**Keywords:** mebendazole, drug delivery, cancer therapy, polymer, HPMA, controlled drug release

## Abstract

Mebendazole and other benzimidazole antihelmintics, such as albendazole, fenbendazole, or flubendazole, have been shown to possess antitumour activity, primarily due to their microtubule-disrupting activity. However, the extremely poor water-solubility of mebendazole and other benzimidazoles, resulting in very low bioavailability, is a serious drawback of this class of drugs. Thus, the investigation of their antitumour potential has been limited so far to administering repeated high doses given peroral (p.o.) or to using formulations, such as liposomes. Herein, we report a fully biocompatible, water-soluble, HPMA copolymer-based conjugate bearing mebendazole (P-MBZ; M_w_ 28–33 kDa) covalently attached through a biodegradable bond, enabling systemic administration. Such an approach not only dramatically improves mebendazole solubility but also significantly prolongs the half-life and ensures tumour accumulation via an enhanced permeation and retention (EPR) effect in vivo. This P-MBZ has remarkable cytostatic and cytotoxic activities in EL-4 T-cell lymphoma, LL2 lung carcinoma, and CT-26 colon carcinoma mouse cell lines in vitro, with corresponding IC_50_ values of 1.07, 1.51, and 0.814 µM, respectively. P-MBZ also demonstrated considerable antitumour activity in EL-4 tumour-bearing mice when administered intraperitoneal (i.p.), either as a single dose or using 3 intermittent doses. The combination of P-MBZ with immunotherapy based on complexes of IL-2 and anti-IL-2 mAb S4B6, potently stimulating activated and memory CD8^+^ T cells, as well as NK cells, further improved the therapeutic effect.

## 1. Introduction

**Figure 1:**
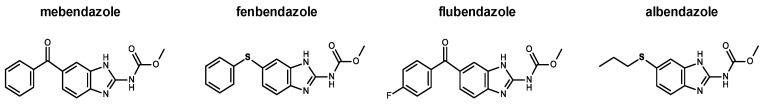
Chemical structures of various benzimidazoles with anticancer activity.

Mebendazole (MBZ) belongs to the group of benzimidazole-based, broad-spectrum antihelmintics, and it has been used for this purpose since 1974, with its antitumour activity being described more than 30 years later [1,2,3,4,5]. MBZ, albendazole, flubendazole, and fenbendazole (Figure 1) are the most promising candidate antitumour agents and exert antitumour activity via the inhibition of tubulin assembly through binding to the colchicine-binding site of β-tubulin, thereby suppressing microtubule formation, followed by mitotic cell arrest and apoptosis [1,3]. The cytotoxic activity of MBZ leads to defective cellular structures, glucose metabolism, and intracellular trafficking [6], as well as Bcl-2 inactivation, causing the increased sensitivity of cancer cells to apoptosis [7]. Consequently, cell proliferation and formation of metastases were inhibited, and synergy with other anticancer drugs (e.g., docetaxel) has been described [1,8,9]. MBZ is also known to inhibit other factors involved in tumour progression, e.g., matrix metalloproteinase-2 activity and angiogenesis [7,10,11,12]. Furthermore, the benzimidazoles are not substrates for P-gp and other ABC-transporters [11], thus avoiding the risk of inducing multidrug resistance in exposed cancer cells, and MBZ reduces P-gp expression.

Several studies have proved the antitumour activity of MBZ in a wide range of cancers, e.g., breast, ovary, lung and colorectal carcinoma, glioblastoma, and melanoma in vitro or in vivo [1,10,13,14]. MBZ demonstrates antitumour activity either as a single agent or in combination with other chemotherapeutics or radiotherapy, inhibiting tumour growth and metastatic spread. Moreover, MBZ also stimulates the antitumour immune response [15,16], but in vivo studies have not fully supported the in vitro data [17,18]. Nevertheless, the extremely poor water-solubility of most benzimidazoles is a serious disadvantage as MBZ is almost completely insoluble in physiologic buffers, thus preventing parenteral administration. Moreover, MBZ has poor bioavailability (~17–20%) upon p.o. administration, with considerable interindividual variability; thus, it is difficult to reach a serum level sufficient for antitumour activity [19,20].

A promising strategy for improving the pharmacologic features of low-molecular-weight drugs, particularly water-solubility and half-life in the circulation, is to employ an advanced drug-delivery system (DDS), such as polymer–drug conjugates, liposomes, or self-assembled micelles [21,22]. The water-soluble, biocompatible polymer carrier based on *N*-(2-hydroxypropyl)methacrylamide (HPMA) is one of the most successful DDSs described so far. Biologically active compounds, typically low-molecular-weight cancerostatic drugs, are covalently attached to this carrier via a defined spacer to achieve controlled release in the target tissue [23,24]. DDSs based on HPMA copolymers significantly reduce the toxicity of the attached drugs, ensuring their increased accumulation in solid tumours via the enhanced permeability and retention (EPR) effect [25,26]. HPMA copolymers bearing different cytostatic drugs have been extensively studied and have proved to possess excellent antitumour activity in numerous mouse and human tumours in vitro and in vivo [27].

Herein, we report the antitumour activities of an HPMA copolymer conjugate bearing MBZ covalently linked to the carrier through a biodegradable bond. The conjugate showed very good water-solubility, enabling administration of MBZ into tumour-bearing mice via i.p. injection, and it showed promising antitumour activity.

## 2. Material and Methods

### 2.1. Synthesis of HPMA Copolymer Conjugate Bearing MBZ

**Figure 2:**
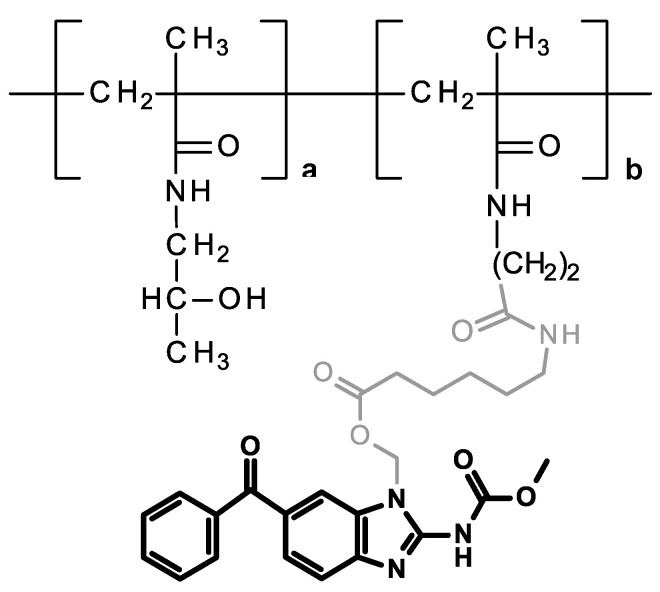
Structure of the HPMA copolymer conjugate bearing MBZ bound through a 6-aminohexanoate-based ester linkage.

The complete synthesis of the HPMA copolymer-bound MBZ has been described recently [28] and involves: (a) derivation of MBZ with a proper functional group enabling linkage to the polymer carrier; (b) preparation of side-chain functionalised HPMA copolymers; (c) and attachment of MBZ derivatives to the polymer precursors yielding the polymer conjugates of interest. In this study, two HPMA copolymer MBZ conjugates, referred to as conjugate **II** in the cited paper [28], were prepared and studied. The MBZ was bound to the polymer carrier via a 6-aminohexanoate-based ester linkage. The schematic structure of the conjugates is shown in Figure 2, and their characteristics are summarised in Table 1. All polymer molecular weights, *M*_n_ and *M*_w_, were measured by gel permeation chromatography (GPC) using a Shimadzu HPLC system equipped with a GPC column (TSKgel G3000SWxl 300 × 7.8 mm; 5 μm), UV–Vis, refractive index (RI) Optilab^®^-rEX and multiangle light scattering (MALS) DAWN EOS (Wyatt Technology Co., Santa Barbara, CA, USA) detector using 80:20 methanol:sodium acetate buffer (pH 6.5; 0.3 M, flow rate 0.5 mL/min). The hydrodynamic diameter (*D_h_*) of the conjugates in PBS buffer (pH 7.4, 5 mg/mL, 25 °C) was measured by a Nano-ZS instrument (ZEN 3600, Malvern, UK). The intensity of scattered light was measured at angle θ = 173°, using a laser with a wavelength of 632.8 nm, and the data were analysed using the DTS (Nano) programme. All values were the mean of five or more independent measurements. The mebendazole content was determined by HPLC after treatment with 1% NaOH solution for 30 min. The analyses were performed via HPLC using a reverse-phase monolithic column (Chromolith Performance RP-18e 100 × 4.6 mm) with UV detection. A mixture of water and acetonitrile was used as an eluent at a gradient of 0–100% and a flow rate of 2.0 mL/min.

### 2.2. Cell Lines

The following murine cancer cell lines from the American Type Culture Collection (ATCC, Manassas, VA, USA) were used: colon carcinoma CT-26, Lewis lung carcinoma LL2, and T-cell lymphoma EL-4. The EL-4 cell line was propagated in RPMI-1640 medium supplemented with 2 mM glutamine, 100 U/mL penicillin, 100 µg/mL streptomycin, 1 mM Na pyruvate, 4.5 g/L of glucose, and 10% heat-inactivated FBS. Dulbecco’s modified eagle medium (DMEM), supplemented with 4 mM glutamine, 100 U/mL penicillin, 100 µg/mL streptomycin, 1.5 g/L Na bicarbonate, 4.5 g/L of glucose, 10 mM of HEPES solution, and 10% heat-inactivated FBS, was used to cultivate the LL2 cells. The CT-26 cell line was propagated in RPMI-1640 medium supplemented with 2 mM glutamine, 100 U/mL penicillin, 100 µg/mL streptomycin, 1 mM Na pyruvate, 4.5 g/L of glucose, 10 mM of HEPES solution, 5 mL of nonessential amino acids, and 10% heat-inactivated FBS. Only cells with viability higher than 95% and within exponential growth were used for experiments. All employed cell lines were used for up to four passages upon thawing. All cell cultures were propagated under standard cultivation conditions (37 °C, 5% CO_2_ humidified atmosphere). Cells were cultivated until reaching ~80–90% confluence before any use. All cell lines were routinely tested for mycoplasma (MycoAlert Mycoplasma Detection Kit, Lonza, Basel, Switzerland).

### 2.3. Mice

C57BL/6 (*H-2^b^*) mice were obtained from the animal facility at the Institute of Microbiology, Czech Academy of Sciences. Food and water were given ad libitum, and the mice were 9–15 weeks of age and weighed 19–22 g. The institutional guidelines for the care and use of laboratory animals were strictly followed in line with a protocol approved by the Institutional Animal Care and Use Committee of the Czech Academy of Sciences for all animal work (Prague, Czech Republic), and the experiments were conducted in compliance with local and European guidelines.

### 2.4. IL-2/S4B6 Complexes

Complexes were prepared by mixing recombinant mouse IL-2 (100 µg/mL; Peprotech, Cranbury, NJ, USA) with anti-IL-2 mAb S4B6 (BioXCell, Lebanon, NH, USA) at a molar ratio of 2:1. After 15 min incubation at room temperature, the complexes were diluted with PBS to the desired concentration before application.

### 2.5. In Vitro Proliferation Assay

To test the in vitro cytostatic activity of the MBZ and polymer conjugates bearing MBZ, inhibition of cell proliferation was determined using the [^3^H]-thymidine incorporation assay. EL-4, LL2, or CT-26 cells (5, 2, and 0.75 × 10^4^/well for 24-, 48-, and 72-h incubation periods, respectively) were seeded in a 96-well, flat-bottom tissue culture plate (Nunc, Roskilde, Denmark). Titrated concentrations of the samples were added to the wells in quadruplicate, to reach a final volume of 250 µL. The plates were incubated in a 5% CO_2_ at 37 °C for 24, 48, or 72 h, and then pulsed with 1 µCi (37 kBq) of [^3^H]-thymidine for the last 6 h of incubation. Next, we harvested the cells on glass fibre filters (PerkinElmer, Waltham, MA, USA) using a cell harvester (Tomtec, Orange, Hamden, CT, USA). A scintillation counter (1450 Microbeta TriLux, Wallac, Turku, Finland) was employed to measure the radioactivity of the samples by the use of plastic scintillator. Control cells were cultivated in a cultivation medium only. The activity of control cells was always higher than 20,000 cpm/well.

### 2.6. In Vitro Cytotoxicity Assay

A conventional MTT (3-(4,5-dimethylthiazol-2-yl)-2,5-diphenyltetrazolium bromide) assay was employed to determine the cytotoxicity of the MBZ and polymer conjugates bearing MBZ in EL-4, LL2, and CT-26 cells in vitro. Tested cells (5, 2, and 0.75 × 10^4^/well for 24, 48, and 72 h incubation periods, respectively) were seeded into 96-well tissue culture plates with flat-bottoms (Nunc, Denmark), and they were cultivated with tested samples for 70 h as described above. Next, plates were centrifuged (200× *g*, 5 min, 4 °C), 200 µL of cultivation medium was aspirated from each well, and 120 µL of MTT (0.83 mg/mL) in culture medium was added to each well. Plates were cultivated for another 2 h at standard cultivation conditions. Finally, 200 µL of DMSO was added to each well, and the absorbance was determined after 15 min at 570 nm using a microplate reader (Infinite 200 PRO, TECAN, Männedorf, Switzerland).

### 2.7. Inhibition of Tumour Growth In Vivo

Female C57BL/6 mice were s.c. inoculated with 1 × 10^5^ EL-4 cells in 100 µL of sterile PBS on the shaved right anterior flank on day 0. Tumour-bearing mice were randomly distributed into experimental groups, and treatment was initiated on day 8 when the tumours reached about 5–7 mm in diameter. The doses were determined based on the mean body weight of each experimental group at the time of drug administration and given in 500 µL, injected i.p. The animals were observed three times a week for tumour progression, and the tumours were measured using callipers. The tumour size, body weight and survival were recorded, with mice that survived until day 80 without any signs of a tumour considered as long-term survivors.

## 3. Results and Discussion

Two polymer conjugates bearing MBZ, namely P-MBZ-A and P-MBZ-B, differing in HPMA copolymer synthesis technique, were synthesised and used in the study. Free radical copolymerisation and reversible addition-fragmentation chain-transfer polymerisation (RAFT) polymerisation techniques were employed. While the free radical polymerisation gives rise to copolymers with rather broad dispersity, RAFT polymerisation enables the synthesis of copolymers with a dispersity close to 1. The conjugate properties are summarised in Table 1, with both polymers having a molecular weight under the threshold limit determined for similar HPMA-based copolymers [29]. Importantly, the hydrodynamic radius significantly increased when the mebendazole derivative was bound to the polymer precursors (from 8.2 to 10.1 nm in the free-radical variant and from 8.6 to 10.3 nm in the RAFT variant), attributed to the steric contribution of a bulky MBZ molecule in the conjugate. Moreover, this is advantageous for an enhanced EPR effect, thus increasing antitumour activity in vivo.

**Figure 3:**
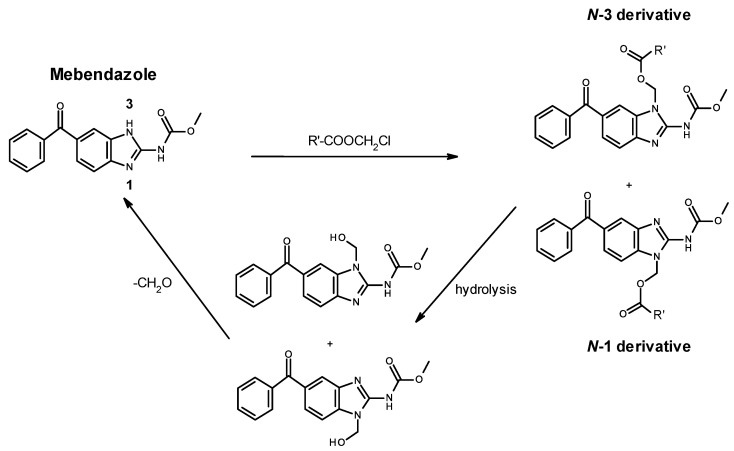
Generation and cleavage of the MBZ derivative. (R’ represents the linker connected to the polymer.) A statistical mixture of *N*-1 and *N*-3 derivatives is formed due to the tautomerism of the MBZ molecule (1,3-H shift).

The linker between the MBZ and polymer carrier was designed to be cleaved by intracellular hydrolases [30], with the hydrolysis of the ester bond within the spacer followed by spontaneous rearrangement of the product releasing the free MBZ (Figure 3).

### 3.1. Cytostatic Activity of P-MBZ Conjugates in Cancer Cell Lines of Various Origins In Vitro

**Figure 4:**
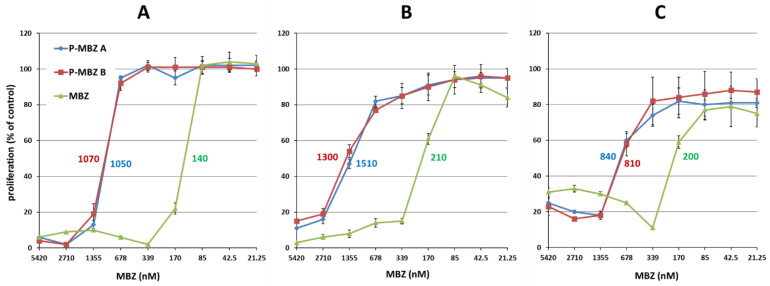
Cytostatic activity of polymer-bound mebendazole conjugates. Cytostatic activity of HPMA copolymer-bound mebendazole conjugates (**P-MBZ-A and P-MBZ-B**) and free mebendazole (MBZ) in EL-4 (**A**), LL2 (**B**) and CT-26 (**C**) murine cancer cell lines in vitro as determined by a [^3^H]-thymidine incorporation assay after 72 h of incubation. The calculated IC_50_ values (nM) are shown inside the graphs for all samples. Each experimental condition was performed in quadruplicate, and the results are shown as the proliferation of exposed cells relative to the controls (untreated cells) ± SD. The experiment was repeated with similar results.

The cytostatic activity of P-MBZ conjugates and free MBZ was first determined in T-cell lymphoma EL-4, lung carcinoma LL2, and colon carcinoma CT-26, with free MBZ showing very similar cytostatic activity in EL-4, LL2, and CT-26 cells, with an IC_50_ of 139, 210, and 200 nM, respectively, after 72 h incubation (Figure 4). Thus, the cytostatic activity of MBZ is comparable to doxorubicin since it has an IC_50_ typically ~75, 130, and 90 nM in these cell lines. This is in concordance with previously reported work showing similar, or even higher, cytostatic activity of MBZ in comparison to other clinically used cancerostatics, e.g., paclitaxel, 5-fluorouracil, oxaliplatin, and others, in different cancer cell lines [11]. **P-MBZ-A** and **P-MBZ-B** conjugates showed practically identical cytostatic activities (Figure 4), which were about 4–8 times less potent than free MBZ (IC_50_ ~ 0.814–1.51 µM). The difference in dispersity between **P-MBZ-A** and **P-MBZ-B** conjugates thus does not affect their in vitro cytostatic activity. The ratio between the cytostatic activities of free MBZ and polymer-MBZ conjugates was similar to that for doxorubicin and the HPMA copolymer conjugate bearing doxorubicin bound through a hydrazone bond [31], demonstrating that MBZ is effectively released from the polymer carrier to its pharmacologically active form. These results demonstrate that polymer-MBZ conjugates possess remarkable cytostatic activity in cancer cell lines of various origins in vitro.

### 3.2. Kinetics of Cytostatic and Cytotoxic Activities of the P-MBZ-A Conjugate and MBZ In Vitro

**Figure 5:**
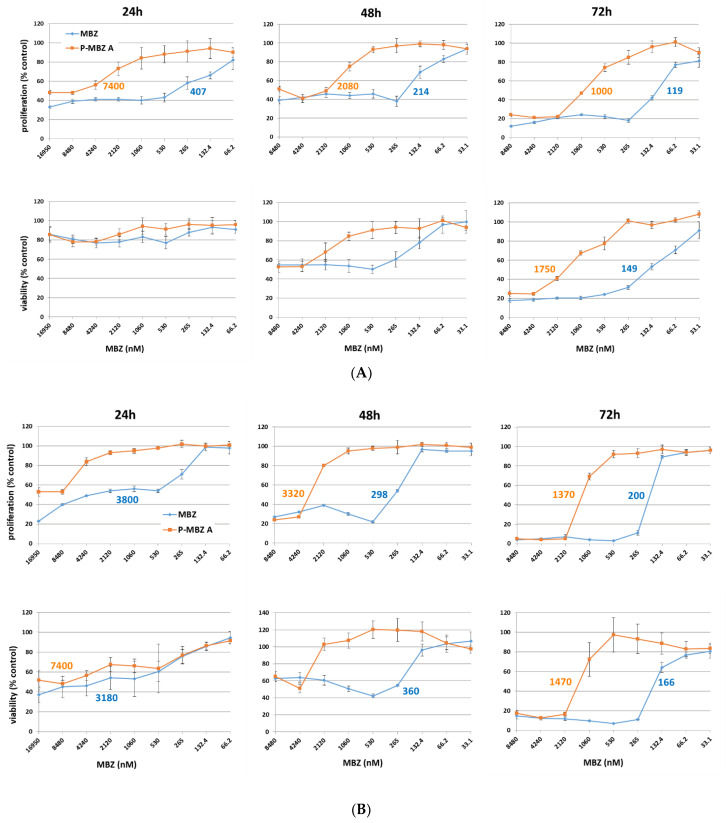
Kinetics of the cytostatic and cytotoxic activities of polymer-bound mebendazole conjugate. Cytostatic activity of HPMA copolymer-bound mebendazole conjugate (**P-MBZ-A**) and free mebendazole (MBZ) in LL2 (**A**) and EL-4 (**B**) murine cancer cell lines in vitro, as determined by [^3^H]-thymidine incorporation and MTT assays, respectively, after 24, 48, and 72 h of incubation. The calculated IC_50_ values (nM) are shown inside the graphs for those samples where these values were reached. Each experiment was performed in quadruplicate, and the results are shown as the proliferation or viability of exposed cells relative to the controls (untreated cells) ± SD. The experiment was repeated with similar results.

The cytostatic and cytotoxic activities of **P-MBZ-A** conjugate and free MBZ were evaluated in LL2 and EL-4 cell lines after 24, 48, and 72 h of incubation (Figure 5), showing that the cytostatic effects of MBZ and **P-MBZ-A** are more rapid in the LL2 cell line than the cytotoxic one at very high concentrations (˃5 µM MBZ). Both MBZ and **P-MBZ-A** inhibited proliferation of LL2 cells to less than 50% of controls after 24 h incubation, with little effect on cell viability. Almost-comparable cytostatic and cytotoxic effects were observed in both cell lines after 48 h of incubation, with **P-MBZ-A** conjugate showing slightly higher cytostatic activity than cytotoxic activity in LL2 cells (IC_50_ being 1 and 1.75 µM, respectively) after 72 h, whereas the cytostatic and cytotoxic activities of **P-MBZ-A** conjugate were comparable in EL-4 cells. These results show that the cytostatic and cytotoxic effects of **P-MBZ-A** conjugate are expressed later in comparison to free MBZ, probably reflecting the release kinetics of the MBZ from the conjugate.

### 3.3. Antitumour Activity of P-MBZ-A Conjugate In Vivo

**Figure 6:**
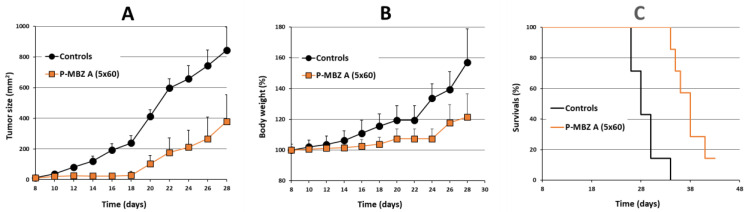
Antitumour activity of prolonged treatment with polymer-bound mebendazole conjugate in vivo. B6 mice (*n* = 7) were s.c. inoculated with 10^5^ EL-4 cells on day 0, then i.p. injected with 5 doses (60 mg mebendazole/kg per one dose) of HPMA copolymer-bound mebendazole conjugate (**P-MBZ-A** (5 × 60)) on days 8, 10, 12, 14, and 16. Untreated mice were used as controls. Tumour growth (**A**), body weight (**B**), and survival (**C**) were monitored, and each experimental point (**A**,**B**) is the average of the experimental group ± SD.

EL-4 tumours growing in syngeneic B6 mice were employed as a model to evaluate the antitumour activity of **P-MBZ-A** as the EL-4 tumour is a rapidly growing, resistant cancer model. First, we evaluated the antitumour activity of **P-MBZ-A** using a prolonged treatment schedule, where P-MBZ-A conjugate was administered i.p. in five doses given every second day. The dose of the conjugate in naïve B6 mice was titrated to determine the safe dose for the selected treatment schedule, which was approximately 60 mg MBZ/kg. B6 mice with progressively growing s.c. EL-4 tumours were treated with **P-MBZ-A** conjugate starting on day 8 post-tumour-cell inoculation (Figure 6), and the treatment was deemed safe as mice did not lose any weight (Figure 6B) or show any other signs of toxicity. **P-MBZ-A** conjugate effectively inhibited tumour growth up to day 18 (Figure 6A), after which the tumours exhibited growth kinetics similar to those of the controls, showing that **P-MBZ-A** was capable of the potent inhibition of tumour progression, but the treatment effect rapidly diminished thereafter. Survival of the mice treated with **P-MBZ-A** was 128% of the controls, with one mouse being completely cured (Figure 6C).

**Figure 7:**
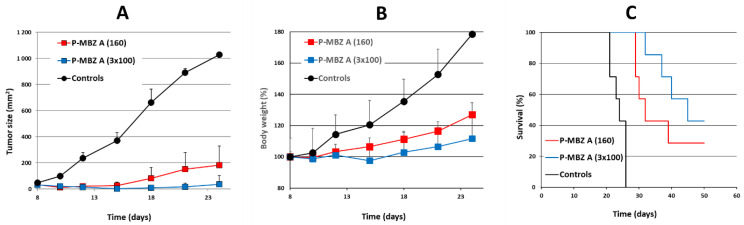
Antitumour activity of short-term treatment with polymer-bound mebendazole conjugate in vivo. B6 mice (*n* = 7) were s.c. inoculated with 10^5^ EL-4 cells on day 0, then i.p. injected, either with 1 dose (160 mg mebendazole/kg) of HPMA copolymer-bound mebendazole conjugate (**P-MBZ-A** (160)) on day 8 or 3 doses (100 mg mebendazole/kg per one dose) of HPMA copolymer-bound mebendazole conjugate (**P-MBZ-A** (3 × 100)) on days 8, 10, and 12. Untreated mice were used as controls. Tumour growth (**A**), body weight (**B**), and survival (**C**) were monitored, and each experimental point (**A**,**B**) is the average of the experimental group ± SD.

Next, the antitumour activity of **P-MBZ-A** conjugate, using a short-term treatment consisting of either a single dose or three doses given every second day, was evaluated. The safe dosage for these treatment schedules was approximately 160 and 100 mg MBZ/kg for a single dose and 3 doses, respectively. B6 mice bearing progressively growing s.c. EL-4 tumours were treated with P-MBZ-A conjugate starting on day 8 post-tumour-cell inoculation (Figure 7). Treated mice did not show any weight loss (Figure 7B) or any other sign of toxicity, with significant inhibition of tumour growth in both treatment groups (Figure 7A), which was considerably longer lasting compared to the previous treatment schedule. A total of 2 out of 7 mice treated with a single dose of P-MBZ-A were completely cured (Figure 7C), and the survival of the remaining mice was prolonged to 133% of the controls. The 3-dose schedule showed the highest efficacy since 3 out of 7 mice were completely cured, and the remaining mice survived an average of 161% longer than the controls.

**Figure 8:**
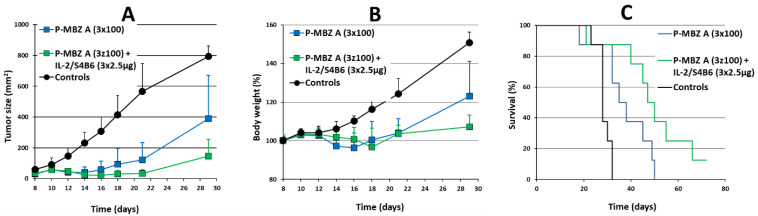
Potentiation of antitumour activity of polymer-bound mebendazole conjugate through IL-2 immunocomplexes in vivo. B6 mice (*n* = 8) were s.c. inoculated with 10^5^ EL-4 cells on day 0, then i.p. injected with 3 doses (100 mg mebendazole/kg per one dose) of HPMA copolymer-bound mebendazole conjugate (**P-MBZ-A** (3 × 100)) on days 8, 10, and 12, or with the latter plus complexes of IL-2 and anti-IL-2 mAb S4B6 mAb (2.5 µg IL-2 per one dose) on days 14, 16, and 18 (**P-MBZ-A** (3 × 100) + IL-2co). Untreated mice were used as controls. Tumour growth (**A**), body weight (**B**), and survival (**C**) were monitored, and each experimental point (**A**,**B**) is the average of the experimental group ± SD.

Finally, we determined whether immunotherapy based on complexes of IL-2 and anti-IL-2 mAb S4B6 (IL-2co) can increase the therapeutic effect of **P-MBZ-A** conjugate. These complexes stimulate the expansion of activated and memory CD8^+^ T-cells, as well as NK cells [32], and have been used previously in combination with HPMA copolymer conjugate bearing doxorubicin, demonstrating significant synergy in terms of antitumour activity in murine BCL1 leukaemia and B16-F10 melanoma. Thus, B6 mice bearing progressively growing s.c. EL-4 tumours were treated with either **P-MBZ-A** conjugate alone or in combination with IL-2co (Figure 8). The conjugate (100 mg MBZ/kg) was administered in 3 doses given on days 8, 10, and 12, while IL-2co was injected on days 14, 16, and 18. No treatment caused significant body weight loss (Figure 8B) or any other sign of toxicity. The treatment with **P-MBZ-A** alone was slightly less effective compared to the previous experiment regarding tumour growth inhibition as well as survival. No mice were completely cured, and survival was only prolonged to 131% of controls. However, P-MBZ-A conjugate in combination with IL-2co showed considerably higher antitumour efficacy, achieving stronger tumour growth inhibition and prolonged survival to 162% of controls. An experimental group treated with IL-2co alone was not included since such immunotherapy given as late as 14 days post-tumour-cell inoculation has no effect [33].

## 4. Conclusions

The linear HPMA copolymer conjugate bearing MBZ covalently attached through a biodegradable bond possesses very good water-solubility, thus enabling parenteral administration, and it has cytostatic activity in the range of 240–450 ng MBZ/mL in several cancer cell lines of various tissue origins in vitro. Of note, the conjugate showed considerable in vivo antitumour activity without any signs of toxicity and could be potentiated through IL-2-based immunotherapy.

## Figures and Tables

**Table 1:** Characteristics of MBZ polymer conjugates and their precursors.

Conjugate	*M*_w_ kDa	Ɖ	Size (*D_h_*) nm	TT/MBZ Content mmol⋅g^−1^	Polymerisation Technique
poly(HPMA-co-Ma-β-Ala-TT) (P-MBZ-A precursor)	28	1.4	8.2	0.33	free radical
poly(HPMA-co-Ma-β-Ala-TT) (P-MBZ-B precursor)	35	1.1	8.6	0.35	RAFT
P-MBZ-A	32	1.6	10.1	0.28	free radical
P-MBZ-B	37	1.1	10.3	0.22	RAFT

## Data Availability

The data presented in this study are available on request from the corresponding author.
